# Supplementary material for: Moving in the Dark—Evidence for an Influence of Artificial Light at Night on the Movement Behaviour of European Hedgehogs (Erinaceus europaeus)
Source: Animals (Basel). 2020 Jul 30;10(8):1306. doi: 10.3390/ani10081306 (PMC7459628; doi:10.3390/ani10081306)
Supplement: Supplementary file 1 [file animals-10-01306-s001.zip › Supplements/Titel_Figures S1-S8.docx]

**Figure Supplement 1: Bootstrap results,** Pictured are the results of the home range estimation using the bootstrap-method for the hedgehogs (A) TR_01_2016, (B) TR_02_2016, (C) TR_08_2016, (D) TR_09_2016, (E) TR_13_2016, and (F) TR_17_2016. The lines indicate the different percentiles of the MCP areas (lower dashed blue line: 0%, lower dotted red line: 25%, continuous black line: 50%, upper dotted red line: 75%, upper dashed blue line: 100%).

**Figure Supplement 2: Bootstrap results.** Pictured are the results of the home range estimation using the bootstrap-method for the hedgehogs (A) TR_19_2016, (B) TR_21_2016, (C) TP_13_2018, (D) TP_18_2018, (E) TP_20_2017, and (F) TP_21_2018. The lines indicate the different percentiles of the MCP areas (lower dashed blue line: 0%, lower dotted red line: 25%, continuous black line: 50%, upper dotted red line: 75%, upper dashed blue line: 100%).

**Figure Supplement 3: Bootstrap results.** Pictured are the results of the home range estimation using the bootstrap-method for the hedgehogs (A) TP_26_2017, (B) TP_28_2017, (C) TP_29_2018, (D) TP_30_2018, (E) TE_02_2019, and (F) TE_03_2019. The lines indicate the different percentiles of the MCP areas (lower dashed blue line: 0%, lower dotted red line: 25%, continuous black line: 50%, upper dotted red line: 75%, upper dashed blue line: 100%).

**Figure Supplement 4: Bootstrap results.** Pictured are the results of the home range estimation using the bootstrap-method for the hedgehogs (A) TE_04_2019, (B) TE_05_2019, (C) TE_07_2019, and (D) TE_10_2019. The lines indicate the different percentiles of the MCP areas (lower dashed blue line: 0%, lower dotted red line: 25%, continuous black line: 50%, upper dotted red line: 75%, upper dashed blue line: 100%).

**Figure Supplement 5: Site fidelity results.** Shown are the site fidelity results for hedgehog TR_01_2016 (A), TR_02_2016 (B), TR_08_2016 (C), TR_09_2016 (D), TR_13_2016 (E), and TR_17_2016 (F). The dashed red lines indicate the interval below which the continuous red line has to be situated to confirm site fidelity for each criterion.

**Figure Supplement 6: Site fidelity results.** Shown are the site fidelity results for hedgehog TR_19_2016 (A), TR_21_2016 (B), TP_13_2018 (C), TP_18_2018 (D), TP_20_2017 (E), and TP_21_2018 (F). The dashed red lines indicate the interval below which the continuous red line has to be situated to confirm site fidelity for each criterion.

**Figure Supplement 7: Site fidelity results.** Shown are the site fidelity results for hedgehog TP_26_2017 (A), TP_28_2017 (B), TP_29_2018 (C), Tp_30_2018 (D), TE_02_2019 (E), and TE_03_2019 (F). The dashed red lines indicate the interval below which the continuous red line has to be situated to confirm site fidelity for each criterion.

**Figure Supplement 8: Site fidelity results.** Shown are the site fidelity results for hedgehog TE_04_2019 (A), TE_05_2019 (B), TE_07_2019 (C), and TE_10_2019 (D). The dashed red lines indicate the interval below which the continuous red line has to be situated to confirm site fidelity for each criterion.
