# Supplementary figures and images for: Moving in the Dark—Evidence for an Influence of Artificial Light at Night on the Movement Behaviour of European Hedgehogs (Erinaceus europaeus)
Source: Animals (Basel). 2020 Jul 30;10(8):1306. doi: 10.3390/ani10081306 (PMC7459628; doi:10.3390/ani10081306)

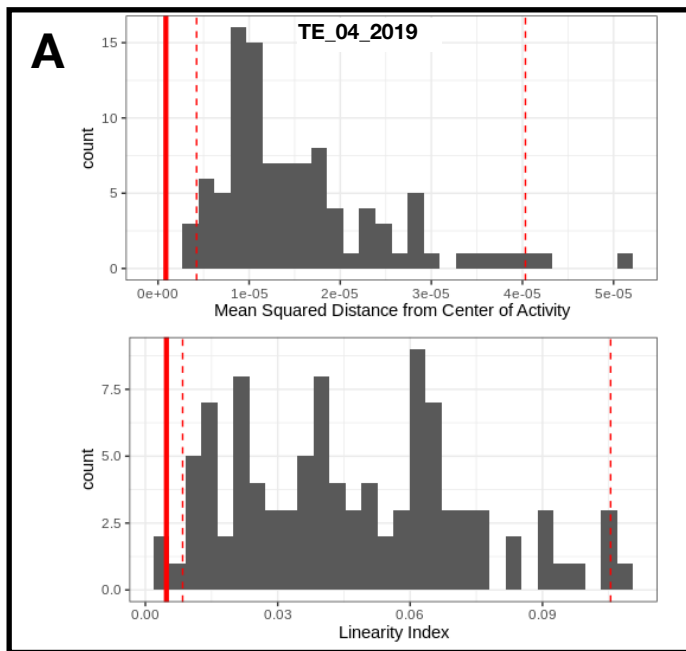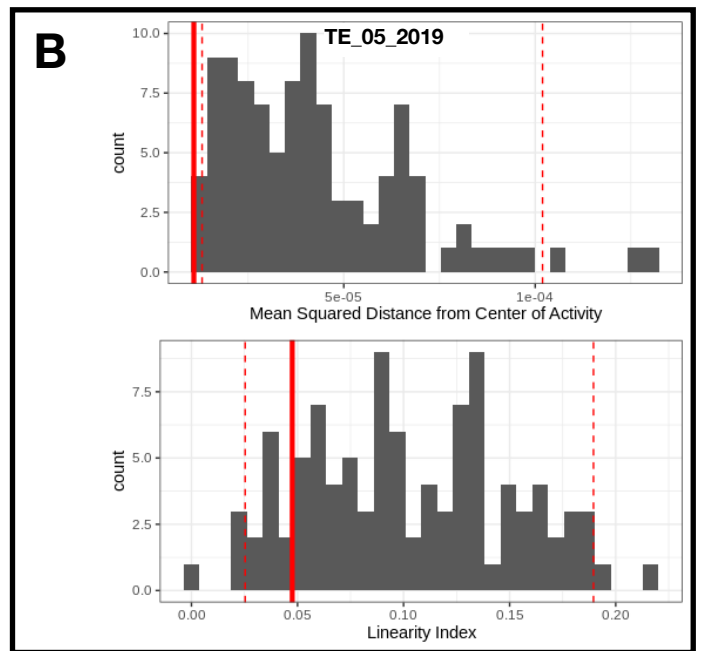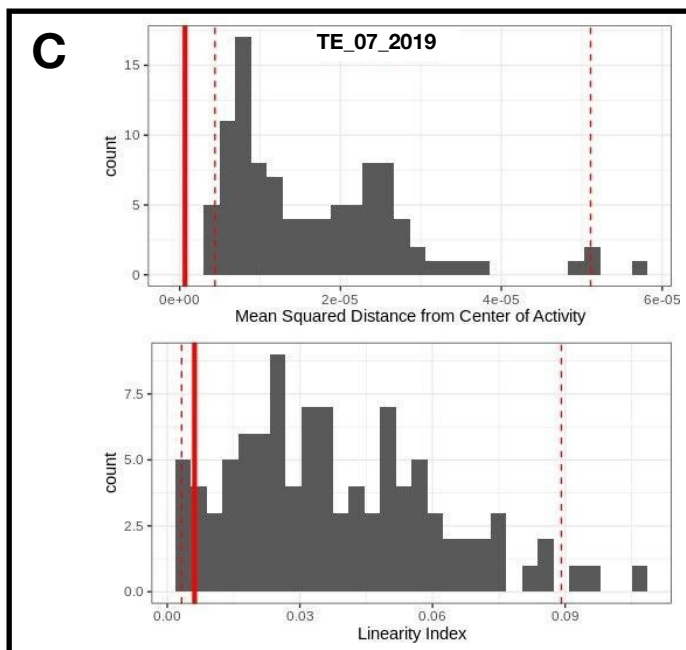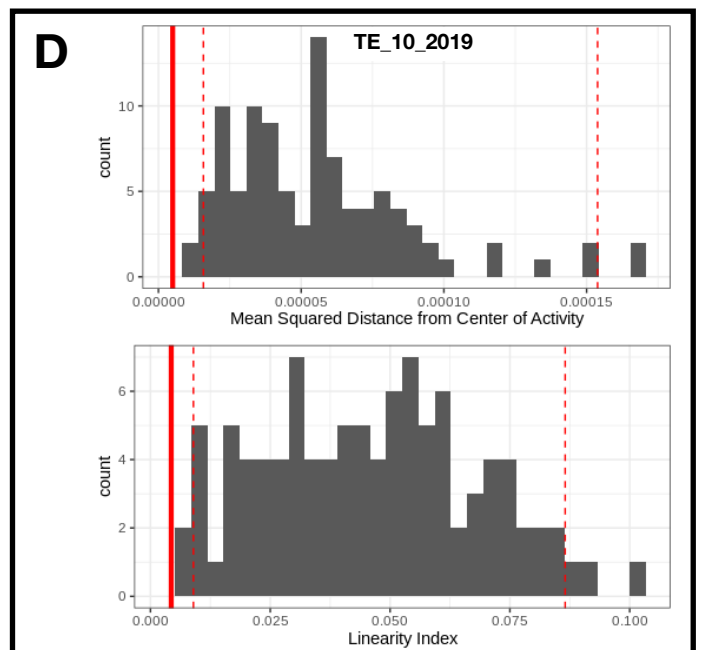

Supplement: Supplementary file 1 [file animals-10-01306-s001.zip › Supplements/Figure_S8.pdf]

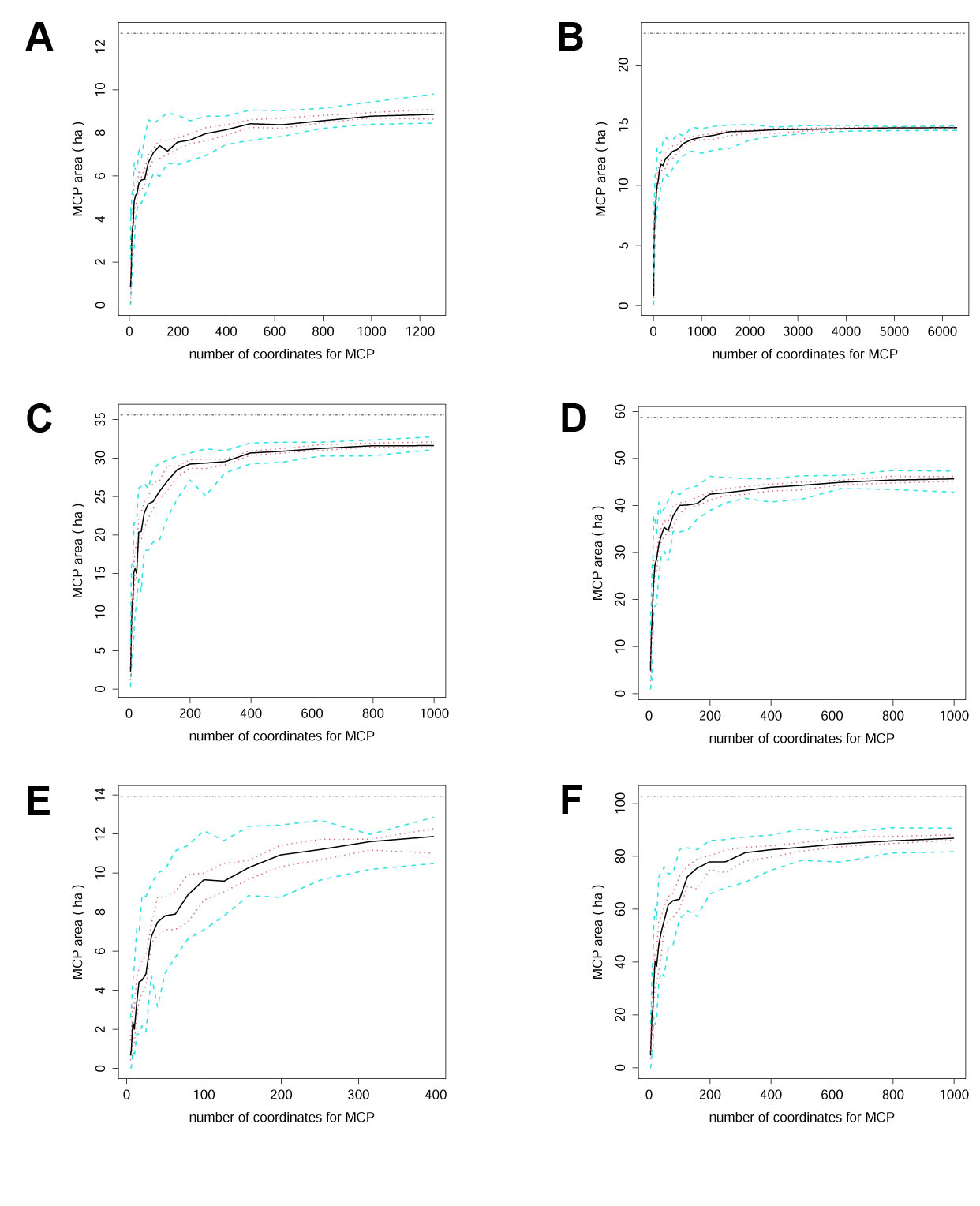

Supplement: Supplementary file 1 [file animals-10-01306-s001.zip › Supplements/Figure_S2.jpg]

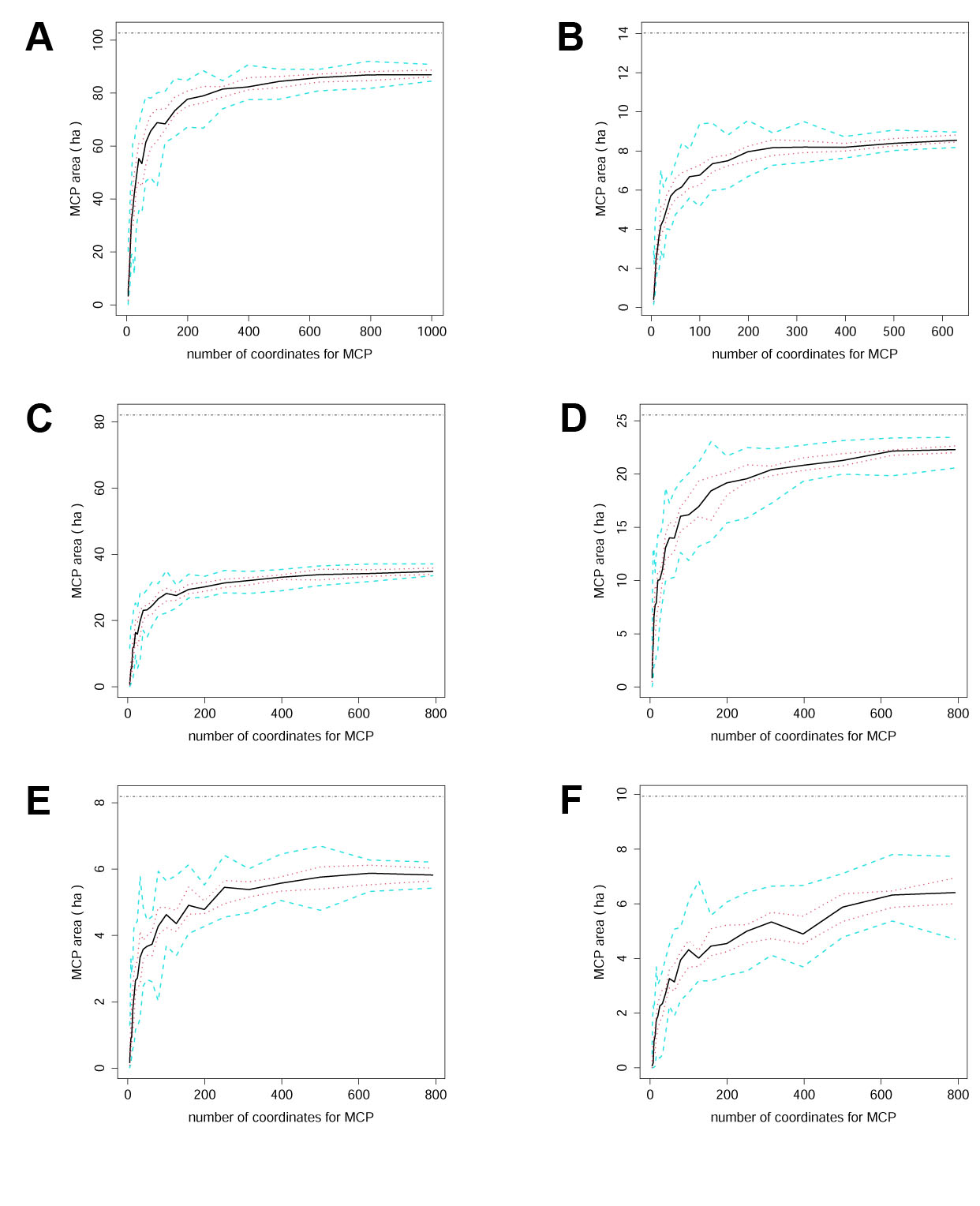

Supplement: Supplementary file 1 [file animals-10-01306-s001.zip › Supplements/Figure_S3.jpg]

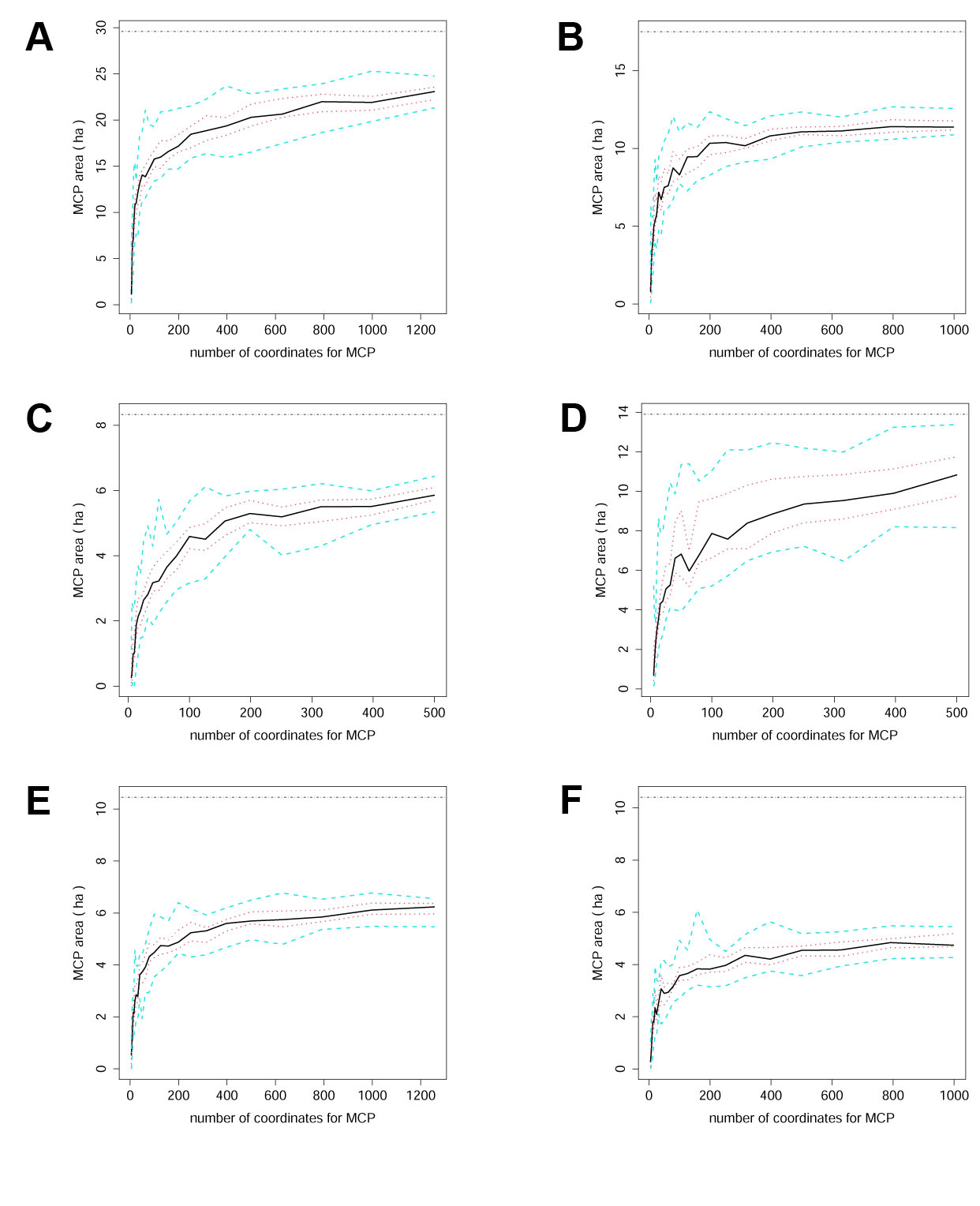

Supplement: Supplementary file 1 [file animals-10-01306-s001.zip › Supplements/Figure S1.jpg]

**A**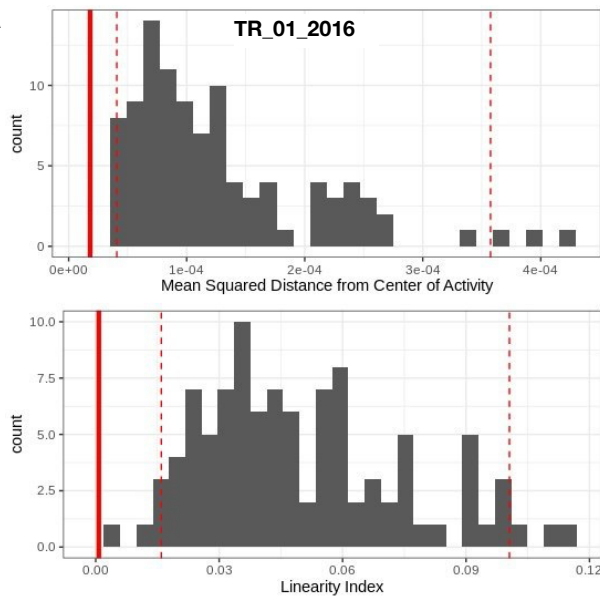**B**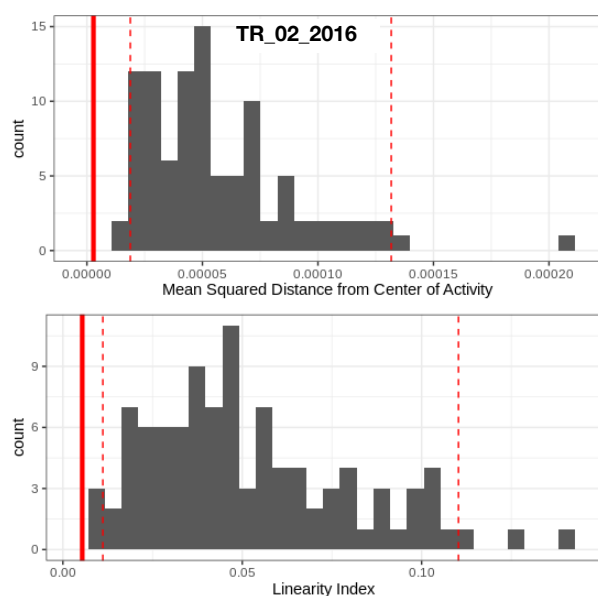**C**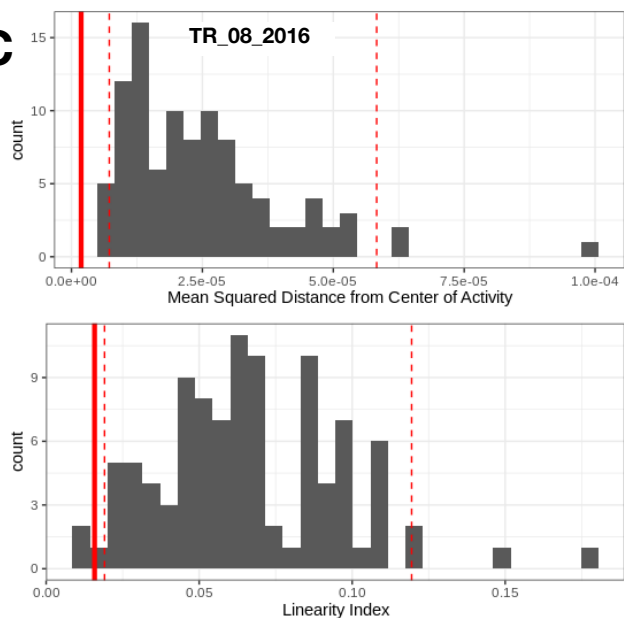**D**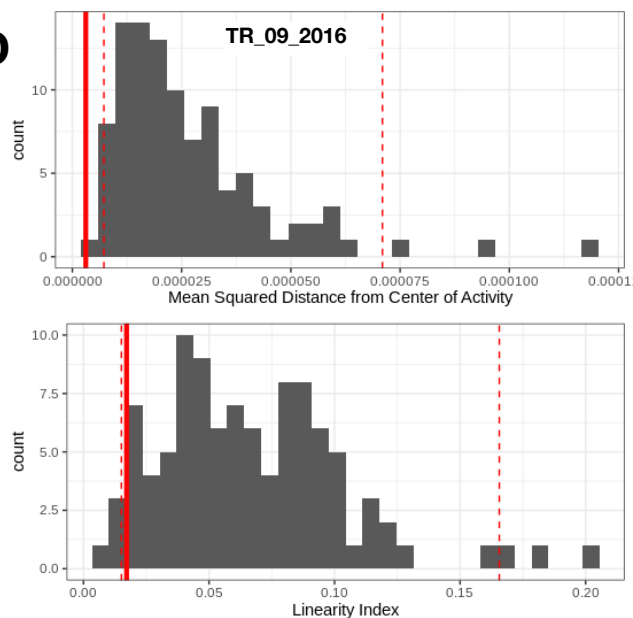**E**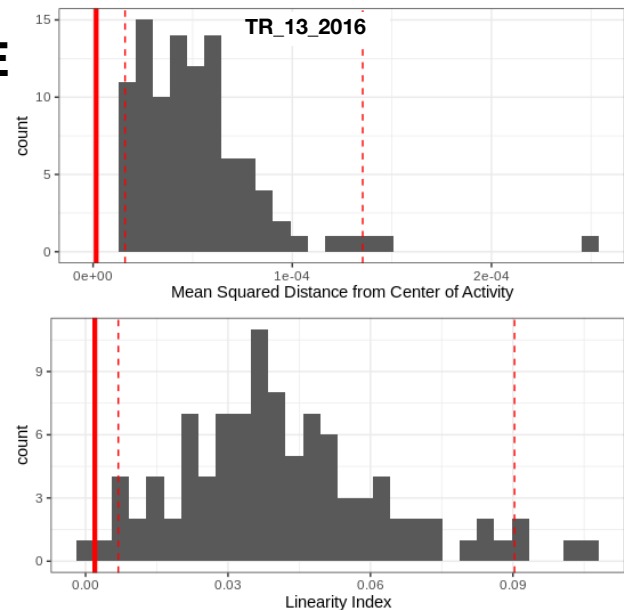**F**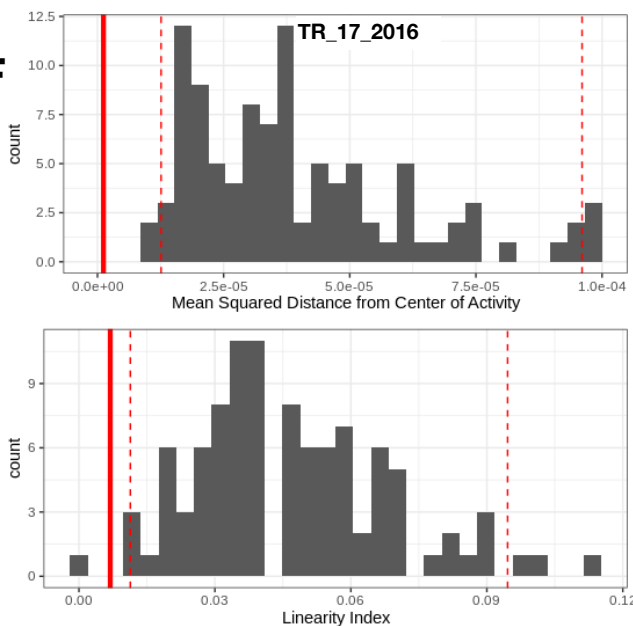

Supplement: Supplementary file 1 [file animals-10-01306-s001.zip › Supplements/Figure_S5.pdf]

**A**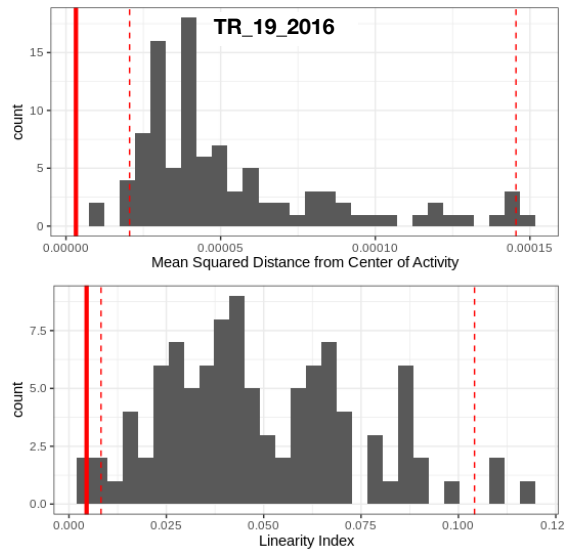**B**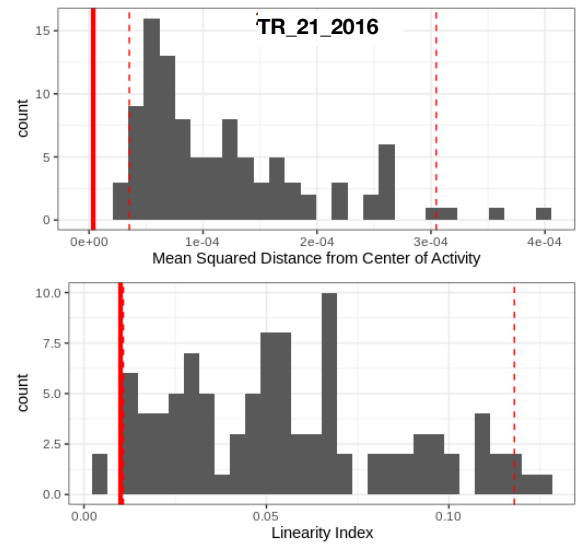**C**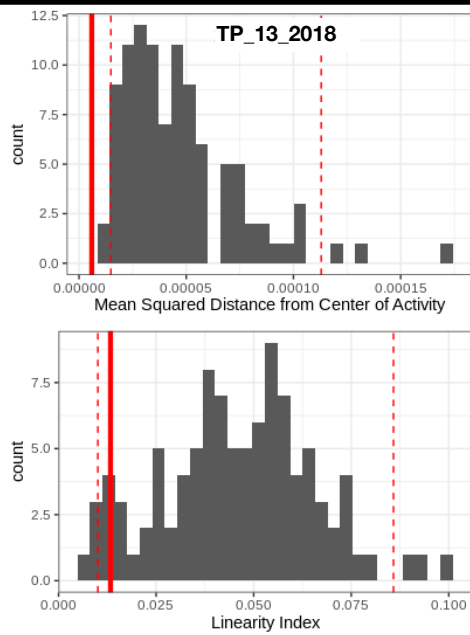**D**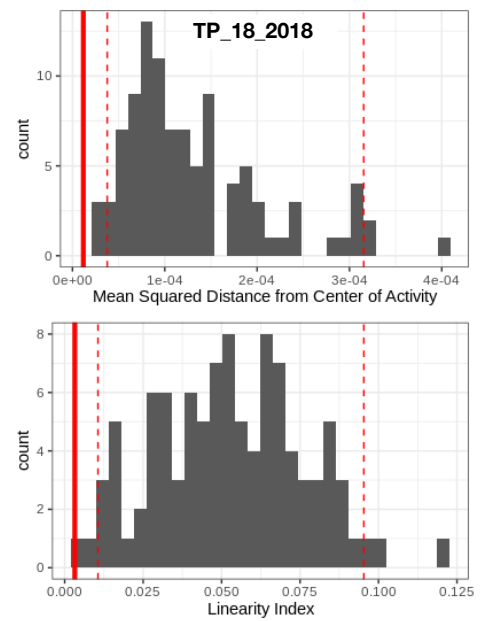**E**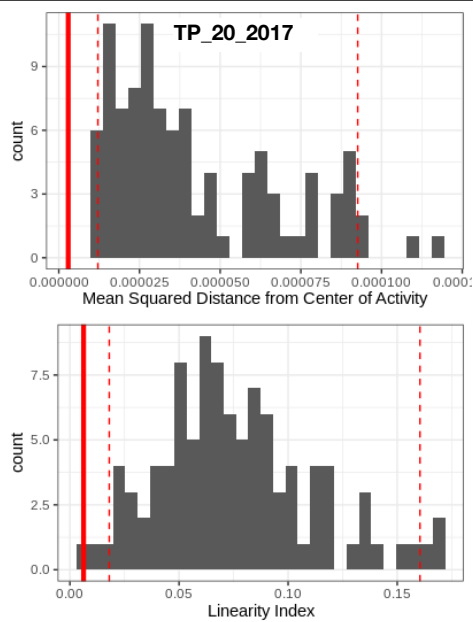**F**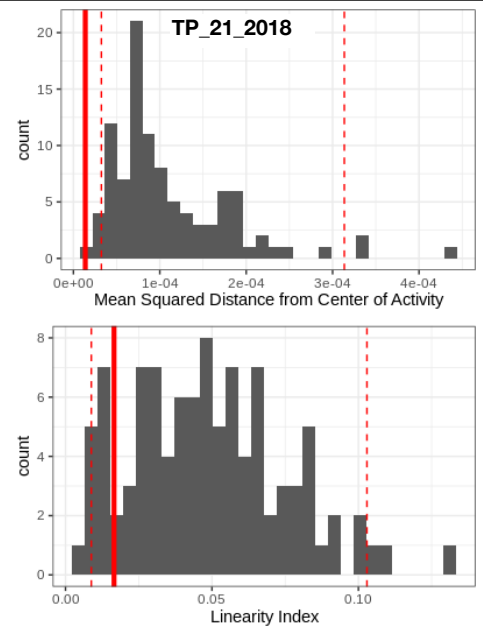

Supplement: Supplementary file 1 [file animals-10-01306-s001.zip › Supplements/Figure_S6.pdf]

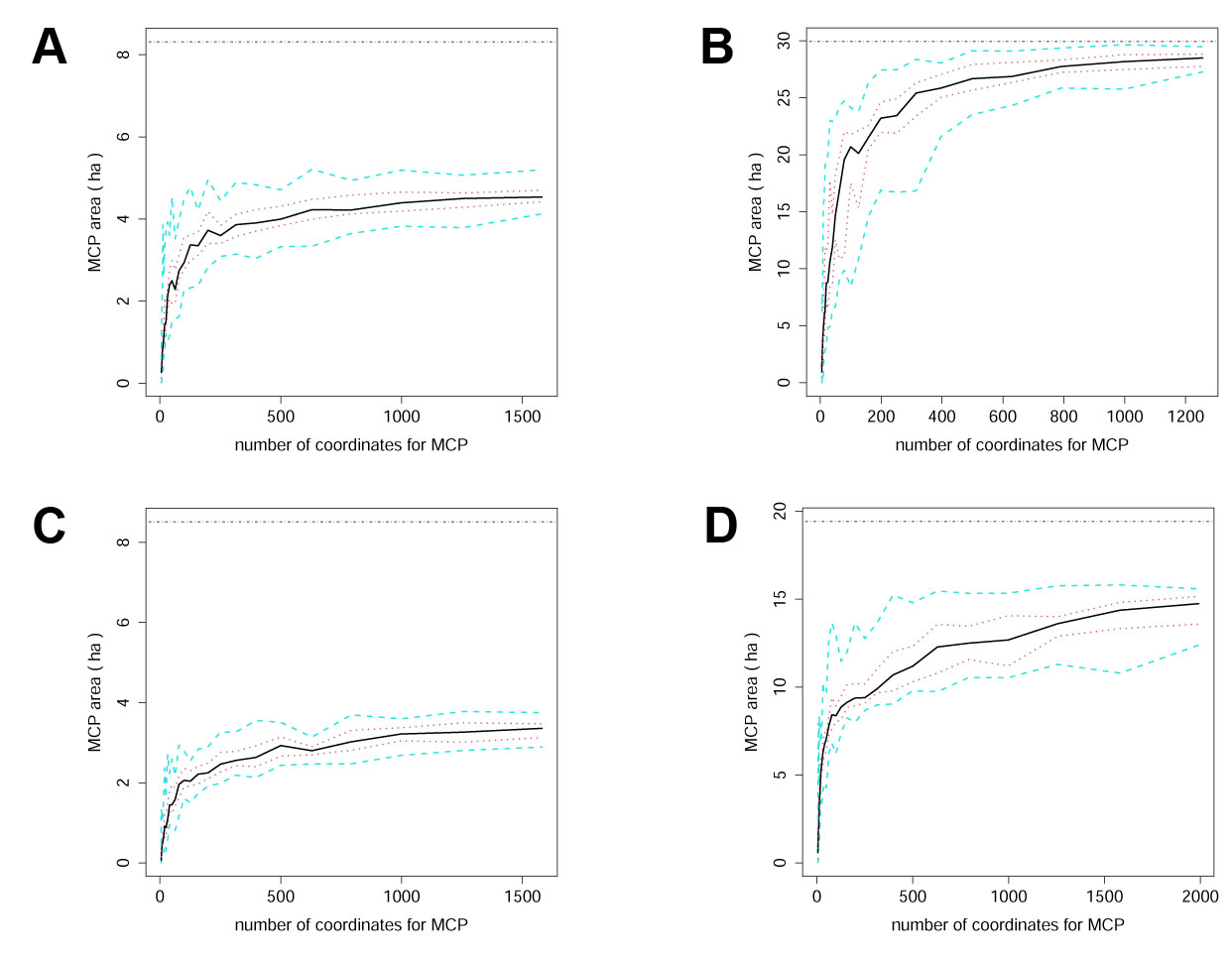

Supplement: Supplementary file 1 [file animals-10-01306-s001.zip › Supplements/Figure_S4.jpg]

**A**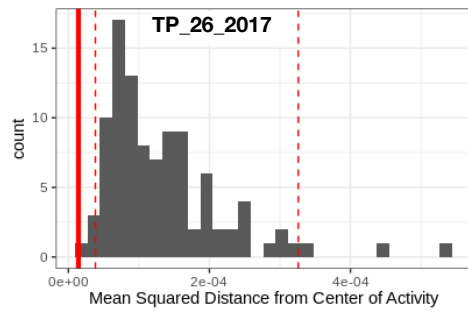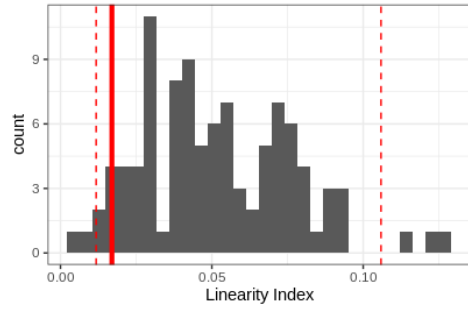**B**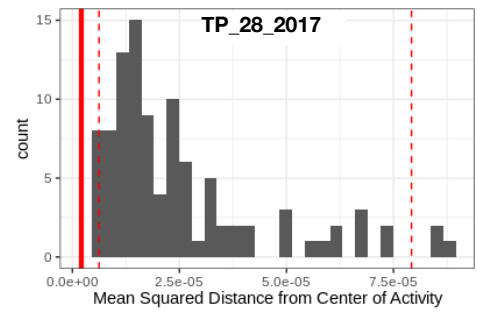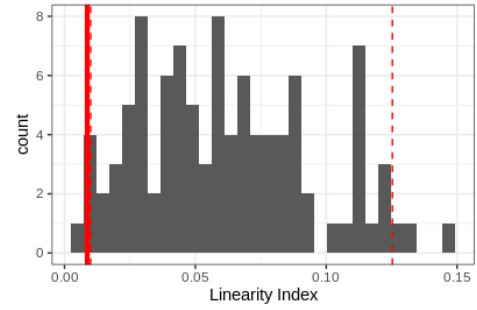**C**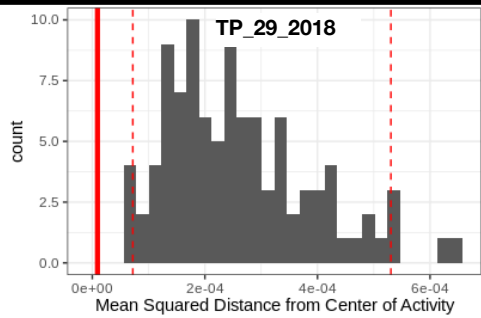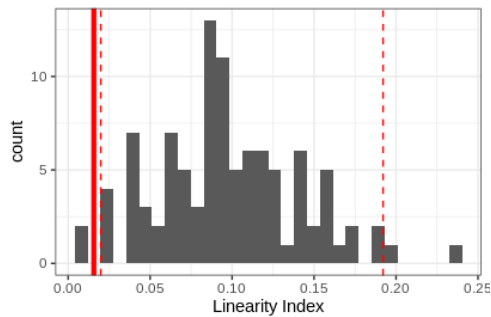**D**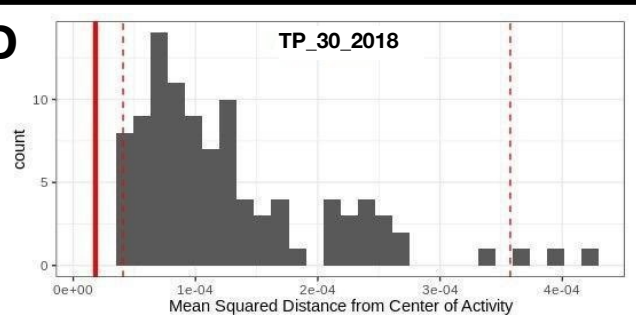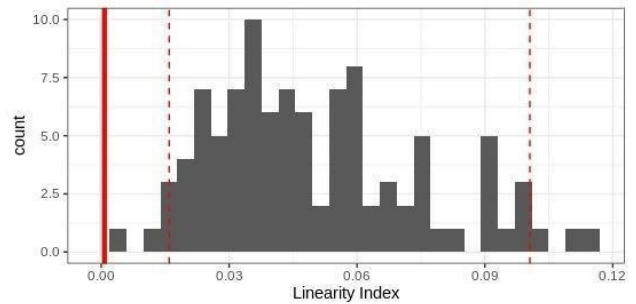**E**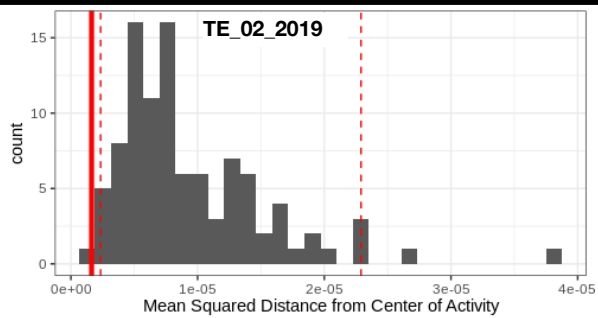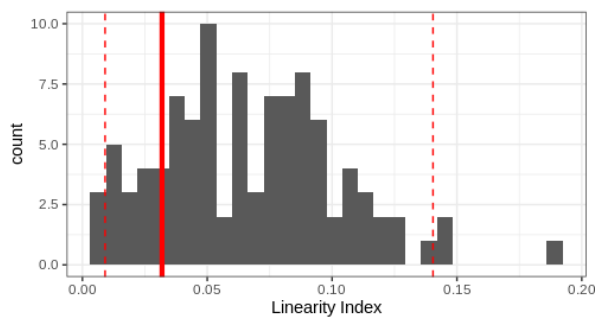**F**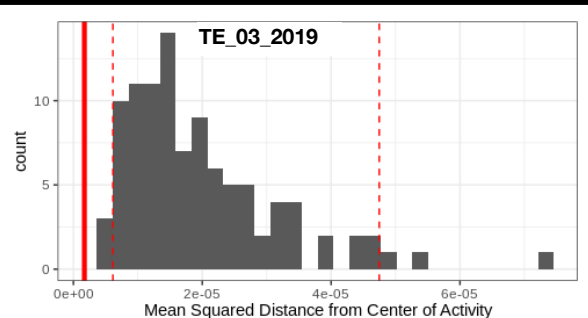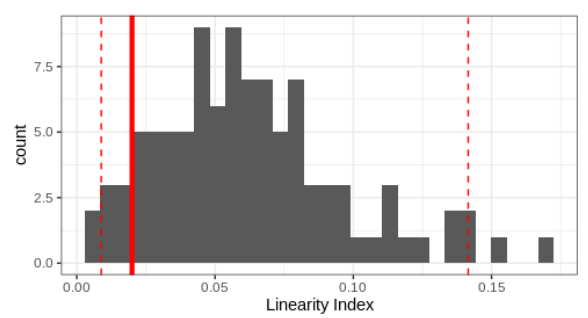

Supplement: Supplementary file 1 [file animals-10-01306-s001.zip › Supplements/Figure_S7.pdf]
